# Cutaneous microcirculation in preterm neonates: comparison between sidestream dark field (SDF) and incident dark field (IDF) imaging

**DOI:** 10.1007/s10877-015-9708-5

**Published:** 2015-05-29

**Authors:** H. A. van Elteren, C. Ince, D. Tibboel, I. K. M. Reiss, R. C. J. de Jonge

**Affiliations:** Division of Neonatology, Department of Pediatrics, Erasmus MC-Sophia Children’s Hospital, University Medical Center, Room Sk-2210, P.O. Box 2060, 3000 CB Rotterdam, The Netherlands; Department of Intensive Care, Erasmus MC, University Medical Center, Rotterdam, The Netherlands; Intensive Care and Department of Pediatric Surgery, Erasmus MC-Sophia Children’s Hospital, University Medical Center, Rotterdam, The Netherlands

**Keywords:** Microcirculation, Preterm neonates, Sidestream dark field, Incident dark field

## Abstract

Incident dark field imaging (IDF) is a new generation handheld microscope for bedside visualization and quantification of microcirculatory alterations. IDF is the technical successor of sidestream dark field imaging (SDF), currently the most used device for microcirculatory measurements. In (pre)term neonates the reduced thickness of the skin allows non-invasive transcutaneous measurements. The goal of this study was to compare the existing device (SDF) and its technical successor (IDF) in preterm neonates. We hypothesized that IDF imaging produces higher quality images resulting in a higher vessel density. After written informed consent was given by the parents, skin microcirculation was consecutively measured on the inner upper arm with de SDF and IDF device. Images were exported and analyzed offline using existing software (AVA 3.0). Vessel density and perfusion were calculated using the total vessel density (TVD) proportion of perfused vessels (PPV) and perfused vessel density. The microcirculation images quality score was used to evaluate the quality of the video images. In a heterogeneous group of twenty preterm neonates (median GA 27.6 weeks, range 24–33.4) IDF imaging visualized 19.9 % more vessels resulting in a significantly higher vessel density (TVD 16.9 vs. 14.1/mm, *p* value < 0.001). The perfusion of vessels could be determined more accurately in the IDF images, resulting in a significant lower PPV (88.7 vs. 93.9 %, *p* value 0.002). The IDF video images scored optimal in a higher percentage compared to the SDF video images. IDF imaging of the cutaneous microcirculation in preterm neonates resulted in a higher vessel density and lower perfusion compared to the existing SDF device.

## Introduction

The invention of handheld microscopes made it possible to visualize the microcirculation of critically ill patients in a non-invasive manner. Since the introduction approximately 15 years ago, several successive devices have been introduced for this purpose. In general, devices consist of an illumination unit for illumination and a light guide with a magnification lens for image transfer to an imaging module such as such as a video camera. Illumination light is green light with a wavelength (548 nm) which ensures optimal absorption of oxyhemoglobin and deoxyhemoglobin thus making it possible to visualize the red blood cell. The surrounding tissue mostly reflects the light, therefore creating contrast. Hereby it is possible to visualize the flowing red blood cells in microcirculation through mucus membranes and directly on the surface of solid organs at a depth of approximately 1 mm.

In 1999, Groner et al. [1] introduced orthogonal polarization spectral (OPS) imaging. The first commercially available device using this OPS technique was called the Cytoscan^®^ (Cytometrics, Philadelphia, USA). OPS imaging uses polarizers to block surface reflection on tissues. The second generation of handheld microscopes was based on sidestream dark field (SDF) imaging. The illumination is provided by surrounding a central light guide with concentrically placed light emitting diodes (LEDs). Hereby the lens is less disturbed by tissue surface reflections [2]. The device gives an analogue output which requires external analogue to digital conversion for software analysis. The MicroScan^®^ was introduced in 2007 by MicroVision Medical (Amsterdam, The Netherlands) and is the most commonly used device for microcirculatory research. A similar device as the Microscan but with a digital output able to connect directly to a lap top computer was introduced by KK Technology called the CapiScope [3].

Videomicroscopy has mainly been used in the adult population and specifically in the intensive care setting. Persistent microcirculatory alterations in the sublingual area has been identified a predictor of adverse outcome [4, 5]. In the pediatric and neonatal population the microcirculation has been studied on a small scale. The small size of patients and lack of cooperation makes it impossible to measure the microcirculation sublingually. In preterm neonates the reduced thickness of the skin allows transcutaneous microcirculatory imaging. In observational studies using OPS imaging, the cutaneous microcirculation was described during transition [6], during erythrocyte transfusion [7] and in term newborns suffering from asphyxia treated with whole body cooling [8]. These studies provided an important insight in the physiology of the microcirculation in newborns. Other studies measured the buccal microcirculation in patients with septic shock [9] or hypoxemic respiratory failure [10, 11]. In line with studies in adults, the microcirculation proved to be predictive for mortality in pediatric patients with sepsis [9].

**Fig. 1:**
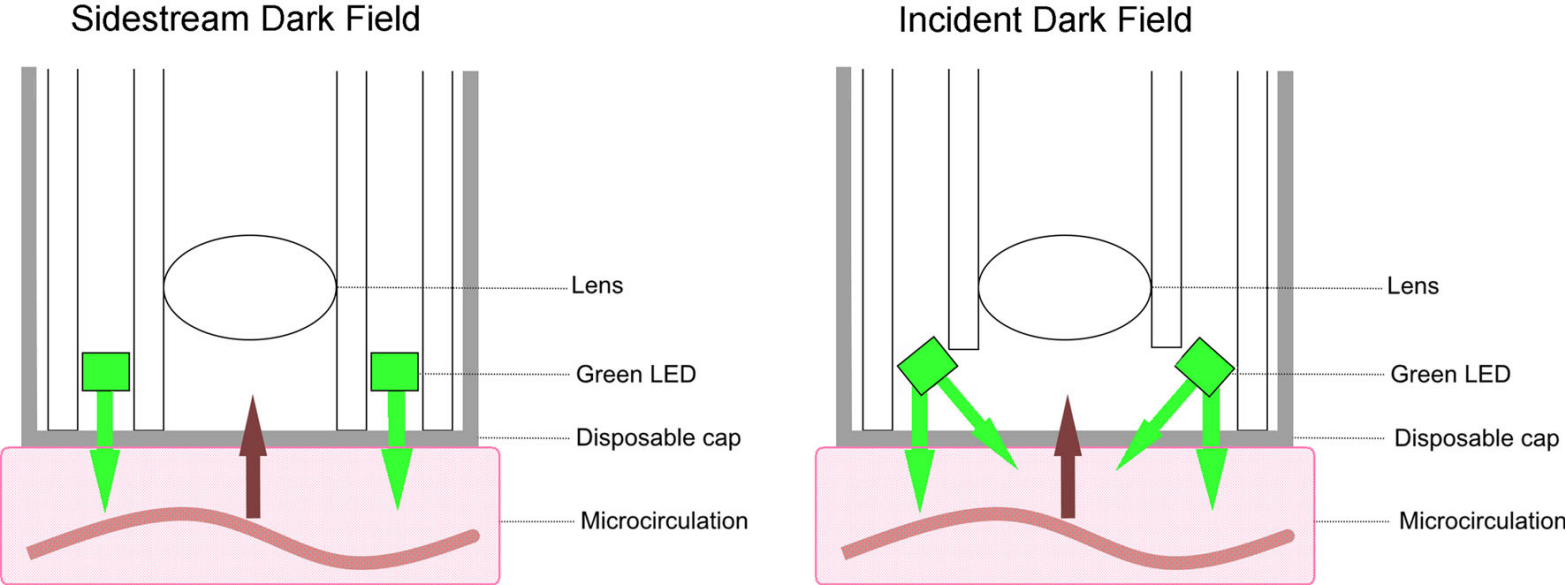
The conceptual differences between SDF and IDF imaging

Recently a third generation handheld microscope has been introduced called the CytoCam^®^ (Braedius Medical, Huizen, The Netherlands). This device is based on incident dark field (IDF) imaging [12]. SDF optically isolates the incoming light from the reflected whilst IDF according to Sherman illuminates the field in a non-homogeneous fashion according to darkfield. Technical improvements, such as digital signal, lower weight of de device and higher optical resolution, have been implemented to overcome persisting limitations of the earlier devices. The technical differences between the SDF and IDF technique are visualized in Fig. 1 and summarized in Table 1. Our group compared the new IDF technique (CytoCam) with the existing SDF technique (MicroScan) in preterm infants. We hypothesized that the technical improvements would result in higher quality images.

**Table 1:** Technical overview of the SDF and IDF devices

	SDF	IDF
Dimensions		
Length (mm)	206	190
Diameter (mm)	64	28
Weight (g)	347	110
Sensor		
Pixel size (µm)	6.25 × 6.25	1.4 × 1.4
Number of megapixel	0.43	14.6
Pulse time (ms)	16	2
Optics		
Resolution (lines per mm)	220	320
Magnification	5	4
Field of view (mm^2^)	0.84	1.79
Focusrange (µm)	0–400	0–400

## Methods

### Study design and setting

This prospective observational study included patients admitted to the neonatal intensive care unit (NICU) of the Erasmus MC-Sophia, a level III university children’s hospital between November and December 2013. The local medical ethical review board approved the study. Parental written informed consent was obtained prior to the study start.

### Patients

In order to obtain a heterogeneous group, the inclusion criteria in terms of gestational age and postnatal age was wide. All hemodynamically stable patients born preterm (GA < 37 weeks) were eligible. An arbitrary cut-off point for postnatal age was set at 6 weeks.

### Microcirculatory imaging

**Fig. 2:**
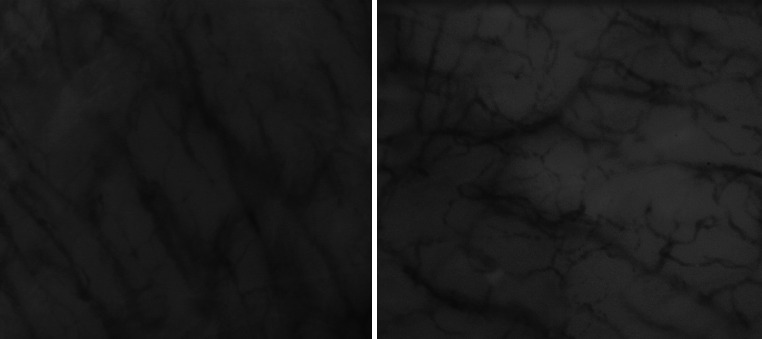
Frames of SDF (*left*) and IDF (*right*) microcirculatory videos

The microcirculation was consecutively measured by SDF imaging (MicroScan) and IDF imaging (CytoCam) at three sites of the upper inner arm. The microcirculation was measured according to the round table guidelines as published by De Backer et al. [13]. All videos were obtained by the same operator. Randomized video sequences were analyzed offline using dedicated software (Automated Vascular Analysis 3.0, Academic Medical Centre, Amsterdam, the Netherlands). Images obtained from the CytoCam were exported in the same video resolution (720 × 580) and surface area as the SDF images (0.94 mm × 0.75 mm). The observer was well-trained and experienced with offline analysis. On all videos, post-process contrast enhancement was applied. Thereafter videos were blinded and anonymized so that the observer was not aware of the used technique. An example of obtained microcirculatory videos is shown in Fig. 2.

Total vessel density (TVD), perfused vessel density (PVD), proportion of perfused vessels (PPV), were calculated for small (Ø ≤ 10 µm) and non-small vessels (Ø 11–100 µm) according to the guidelines [13]. For determining the microvascular flow index (MFI) and heterogeneity index (HI), each video sequence was divided in four equally sized quadrants. Per quadrant the predominant type of flow was scored. MFI represented the mean score of the predominant type of flow, and HI represented the difference between the highest quadrant and the lowest quadrant score that is then divided by the mean score of all quadrants for one measurement. For all other scores, the average of the three video sequences per measurement was taken. To quantify the quality of the videos, the microcirculation imaging quality score was applied [14]. This score is based on six common image capture and analysis problem areas: illumination, duration, focus, content, stability and pressure. Each category is scored as optimal (0 points), suboptimal but acceptable (1 point) or unacceptable (10 points).

### Statistical analysis

Normal distribution of the population could not be assumed. Hereby the groups were compared using the Wilcoxon signed rank test for paired continuous data. For comparison of categorical data the Chi squared test was used. Statistics were calculated using IBM SPSS 21. A two sided *p* value <0.05 was considered statistically significant. A Bland–Altman plot was created with the mean bias and 95 % limits of agreement.

## Results

During the study period, a total of twenty patients were enrolled resulting in 60 video images per device. Table 2 shows the demographic data of the included patients. All patients were hemodynamically stable. Five patients (25 %) were mechanically ventilated. Rectal temperature was between 36.5 and 37.5 degrees Celsius in all patients. Video images were obtained at a median postnatal age of 5 days (range 0–41 days).

**Table 2:** Demographic data of the population (n = 20)

Male Gender (%)	50
Gestational age (weeks)	27.6 (24–33.4)
Gestational age (weeks)	27.6 (24–33.4)
Birth weight in grams	1117 (470–2650)
Apgar score at 1 min	6 (1–9)
Apgar score at 5 min	8 (3–10)
Caesarean Section (%)	45
Antenatal corticosteroids (%)	75

Data are presented as median (range) or percentage

**Fig. 3:**
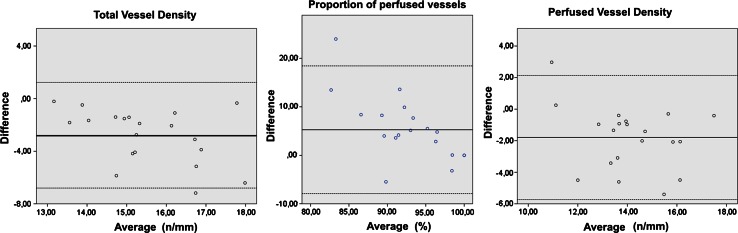
Bland-Altman for TVD, PPV and PVD comparing SDF with IDF

The IDF technique visualized significantly more vessels which resulted in a higher TVD (mean 16.9 vs. 14.1/mm *p* < 0.001). This was mainly because more small vessels were visualized. The PPV was lower in the IDF group versus the SDF group. This was seen in both small as non-small vessels. The differences in TVD and PPV also resulted in a significant higher PVD for small and total vessels (Table 3). Figure 3 shows Bland–Altman plots of the TVD, PPV and PVD. It demonstrates that the SDF structurally measures a lower TVD and PVD and a higher PPV (mean bias TVD −2.8 95 % limits of agreement 1.2 to −6.9; mean bias PPV 5.3, 95 % limits of agreement 18.4 to −7.9; mean bias PVD −1.8, 95 % limits of agreement 2.1 to −5.7)

**Table 3:** Mean microcirculatory parameters total vessel density (TVD), perfused vessel density (PVD), proportion of perfused vessels (PPV), microvascular flow index (MFI) and heterogeneity index (HI) for small and non-small vessels measured by IDF and SDF

	IDF (SD)	SDF (SD)	Change (%)	*p* value*
TVD small (n/mm)	14.8 (2.1)	12.5 (1.8)	+18.4	.001
TVD non-small (n/mm)	2.1 (1.1)	1.6 (1.1)	+31.3	.070
TVD total (n/mm)	16.9 (2.0)	14.1 (1.3)	+19.9	.000
PPV small (%)	88.0 (9.4)	93.6 (7.6)	−5.6	.003
PPV non-small (%)	95.1 (8.4)	98.9 (2.2)	−3.8	.023
PPV total (%)	88.7 (9.1)	93.9 (7.4)	−5.2	.002
PVD small (n/mm)	13.0 (2.1)	11.6 (1.9)	+12.1	.033
PVD non-small (n/mm)	2.0 (1.1)	1.6 (1.1)	+25.0	.103
PVD total (n/mm)	15.0 (2.2)	13.2 (1.7)	+13.6	.001
MFI small (au)	2.78 (0.3)	2.75 (0.3)	+1.1	.621
MFI non-small (au)	3.00 (–)	3.00 (–)	–	–
HI small (au)	0.22 (0.19)	0.24 (0.18)	−8.3	.650
HI non-small (au)	0.00 (–)	0.00 (–)	–	–

au = arbitrary units

* Wilcoxon signed rank test for paired continuous data

Table 4 shows the microcirculation imaging quality score. In the majority of the video clips, the duration was below 3 s and therefore scored 10 points. However, the stability of the video clips was optimal (0 points). This was for both the IDF as the SDF device. In the categories focus and pressure, the majority of the IDF videos were optimal (52/60) while the SDF video clips scored suboptimal (1 point) in a much higher rate (37/60). This difference was highly significant with a *p* value below .001. The percentage of artefacts in the category content was equal.

**Table 4:** The microcirculation image quality score for IDF (n = 60) and SDF (n = 60) video images

	Device	Score 0	Score 1	Score 10	*p* value*
Illumination	IDF	56	4	0	.032
	SDF	48	12	0	
Duration	IDF	5	5	50	.238
	SDF	6	11	43	
Focus	IDF	52	8	0	<.001
	SDF	20	37	3	
Content	IDF	44	16	0	.838
	SDF	43	17	0	
Stability	IDF	59	3	0	.648
	SDF	58	2	0	
Pressure	IDF	59	1	0	<.001
	SDF	44	16	0	

* Chi squared test for categorical data

## Discussion

This study demonstrates that the IDF device visualized approximately 20 % more vessels and therefore provides a higher TVD than the SDF device. The increased vessel density visualized with the IDF device can be explained by the improved imaging technology. Compared with the SDF device, the IDF device signal is fully digital and contains a high resolution sensor. A shortened pulse time (2 vs. 16 ms) creates more contrast and contours of the moving red blood cells. The ergonomic improvements are probably even more important for the higher quality of the images. The low weight of the device (120 vs. 350 g) minimizes pressure artefact problems. The focus control is integrated in the computer software. This adjustment makes it easier to determine the accurate focus and avoids disturbance of camera handling during recording. These improvements are likely to cause the differences in microcirculation images quality scores (Table 4). Especially in the category focus, the IDF device produce higher quality video images. This also applies to the category of pressure. The difference in PPV is therefore likely to be caused by the quality of the images. Due to the suboptimal focus mechanism in the SDF device, it sometimes may be difficult to accurately judge the flow pattern and the presence of pressure artefacts. In a healthy patient the flow is most likely to be undisturbed. In case of doubt, the flow in specific vessels may therefore more likely to be judged positive.

To our best knowledge, this is the first paper reporting a comparison between two microcirculation imaging techniques in preterm infants. Second, it is the first paper presenting the microcirculation quality scoring system for cutaneous microcirculatory images. The scoring system was based on the experience of investigators of the sublingual microcirculation in an adult population. We fully support this effort of quantifying and improving the research field of the microcirculation. However, in the field of pediatric and neonatal intensive care, most of the patients are non-sedated and non-cooperative and therefore the sublingual area is not feasible for microcirculatory research. The buccal and cutaneous microcirculation are good alternatives but the lack of patient cooperation still makes it a challenge to obtain microcirculatory images that fully meet the criteria of the microcirculation image quality score, especially in the category ‘duration’. Obviously, duration and stability of the video images go hand-in-hand. The majority of our video images were perfectly stable but below 3 s of length (10 points) and according to the scorings system would be unacceptable for analysis. In our view, the shorter length of the video images is inherent in performing microcirculatory research in neonates and infants. The other five categories of the scorings system is fully applicable to buccal or cutaneous video images.

It must be said that the compared videos are not measured on the exact same spot as the skin of preterm infants is too vulnerable for marking. The comparison of the two devices is therefore not based on completely identical video clips. Also, in the majority of the cases only three movies where used for analysis. Since three movies is reliable for analysis [13], we did not want to burden the preterm neonates further, as incubator- and thereby body temperature can decrease to increased measurement time. We do however think the results of this study reflect the differences between the devices as the measured vessel density was structurally higher in the IDF device compared to the SDF device.

## Conclusion

The IDF imaging device represents the third generation hand held microscope visualizing the microcirculation. Technical and ergonomic improvements, in comparison with its predecessor SDF imaging, provides higher quality images of cutaneous microcirculation in preterm infants. This resulted in a higher TVD count and a more accurate judgment of the red blood cell flow and PPV, making it a promising technique for the use of microcirculatory observations in (preterm) newborns.
